# Supplementary material for: Bayesian refinement of protein structures and ensembles against SAXS data using molecular dynamics
Source: PLoS Comput Biol. 2017 Oct 18;13(10):e1005800. doi: 10.1371/journal.pcbi.1005800 (PMC5662244; doi:10.1371/journal.pcbi.1005800)
Supplement: S1 Archive — (BZ2) [file pcbi.1005800.s011.tar.bz2 › gromacs_4.62_bayesian_saxs_refi/share/html/online/tpbconv.html]

tpbconv


| tpbconv Main Table of Contents |

**VERSION 4.6  
Sat 19 Jan 2013**

---

### Description

tpbconv can edit run input files in four ways.

**1.** by modifying the number of steps in a run input file
with options -extend, -until or -nsteps
(nsteps=-1 means unlimited number of steps)

**2.** (OBSOLETE) by creating a run input file
for a continuation run when your simulation has crashed due to e.g.
a full disk, or by making a continuation run input file.
This option is obsolete, since mdrun now writes and reads
checkpoint files.
**Note** that a frame with coordinates and velocities is needed.
When pressure and/or Nose-Hoover temperature coupling is used
an energy file can be supplied to get an exact continuation
of the original run.

**3.** by creating a .tpx file for a subset of your original
tpx file, which is useful when you want to remove the solvent from
your .tpx file, or when you want to make e.g. a pure Cα .tpx file.
Note that you may need to use -nsteps -1 (or similar) to get
this to work.
**WARNING: this .tpx file is not fully functional**.

**4.** by setting the charges of a specified group
to zero. This is useful when doing free energy estimates
using the LIE (Linear Interaction Energy) method.

### Files

| option | filename | type | description |
| --- | --- | --- | --- |
| **-s** | topol.tpr | Input | Run input file: tpr tpb tpa |
| **-f** | traj.trr | Input, Opt. | Full precision trajectory: trr trj cpt |
| **-e** | ener.edr | Input, Opt. | Energy file |
| **-n** | index.ndx | Input, Opt. | Index file |
| **-o** | tpxout.tpr | Output | Run input file: tpr tpb tpa |

### Other options

| option | type | default | description |
| --- | --- | --- | --- |
| **-[no]h** | bool | no | Print help info and quit || **-[no]version** | bool | no | Print version info and quit || **-nice** | int | 0 | Set the nicelevel || **-extend** | real | 0 | Extend runtime by this amount (ps) || **-until** | real | 0 | Extend runtime until this ending time (ps) || **-nsteps** | int | 0 | Change the number of steps || **-time** | real | -1 | Continue from frame at this time (ps) instead of the last frame || **-[no]zeroq** | bool | no | Set the charges of a group (from the index) to zero || **-[no]vel** | bool | yes | Require velocities from trajectory || **-[no]cont** | bool | yes | For exact continuation, the constraints should not be applied before the first step || **-init\_fep\_state** | int | 0 | fep state to initialize from |

---

http://www.gromacs.org  
gromacs@gromacs.org
